# Supplementary figures and images for: SFSWAP is a negative regulator of OGT intron detention and global pre-mRNA splicing
Source: eLife. 2025 Apr 23;13:RP104439. doi: 10.7554/eLife.104439 (PMC12017769; doi:10.7554/eLife.104439)

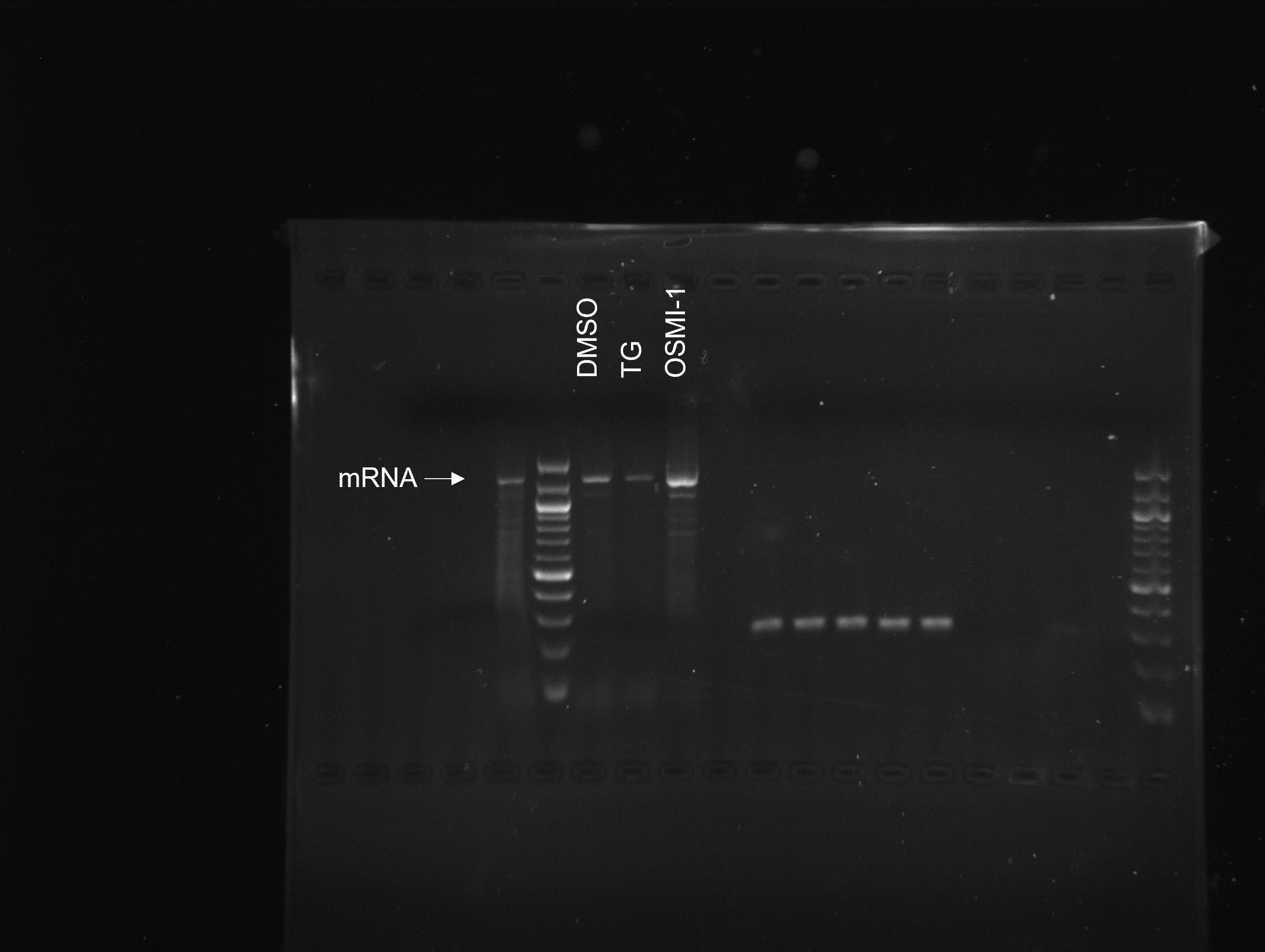

Supplement: Figure 1—source data 1. [file elife-104439-fig1-data1.zip › Figure 1-Source Data 1/Figure 1-Source Data 1-labeled.tif]

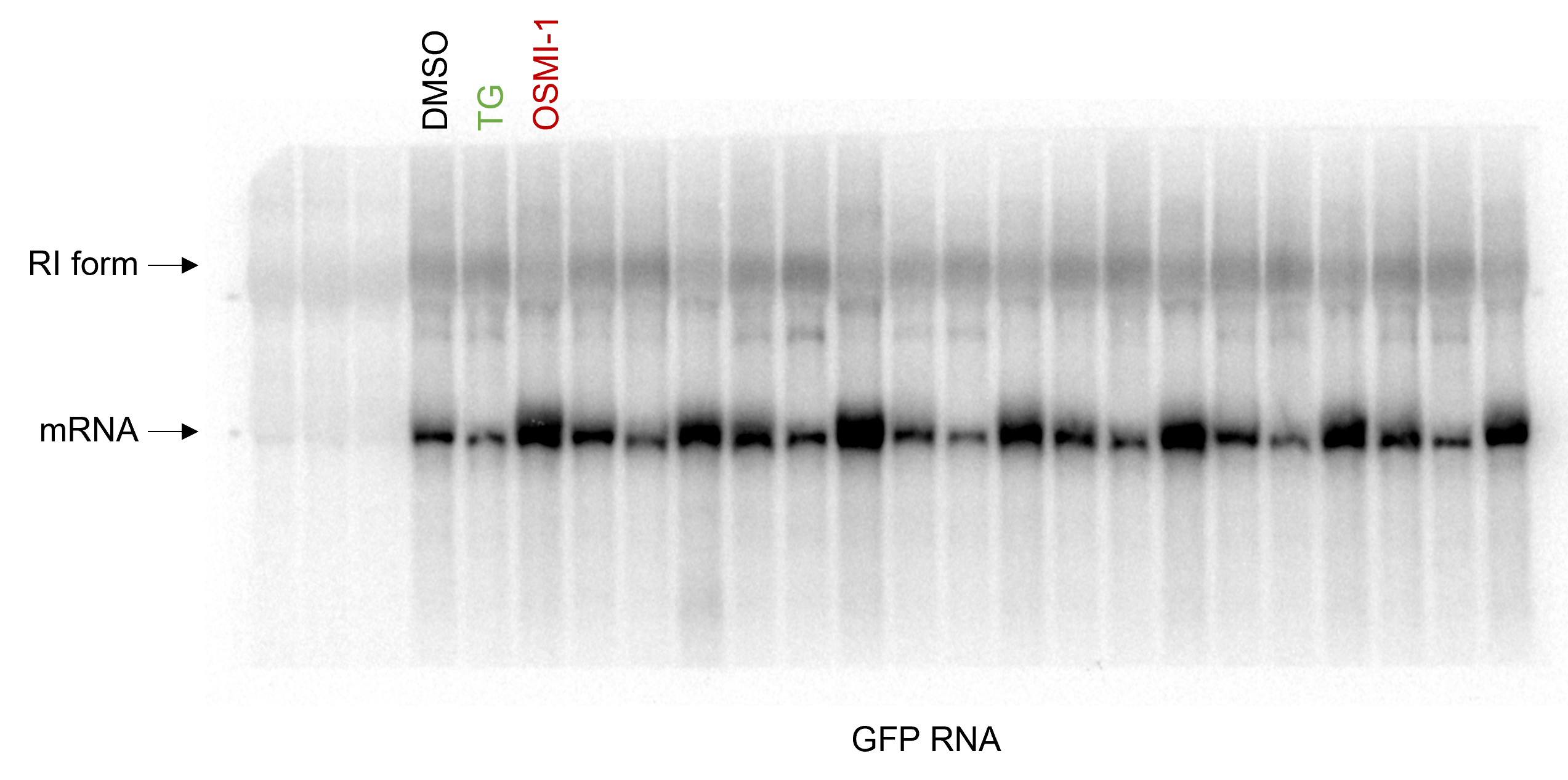

Supplement: Figure 1—source data 2. [file elife-104439-fig1-data2.zip › Figure 1-Source Data 2/Figure 1-Source Data 2-labeled.tif]

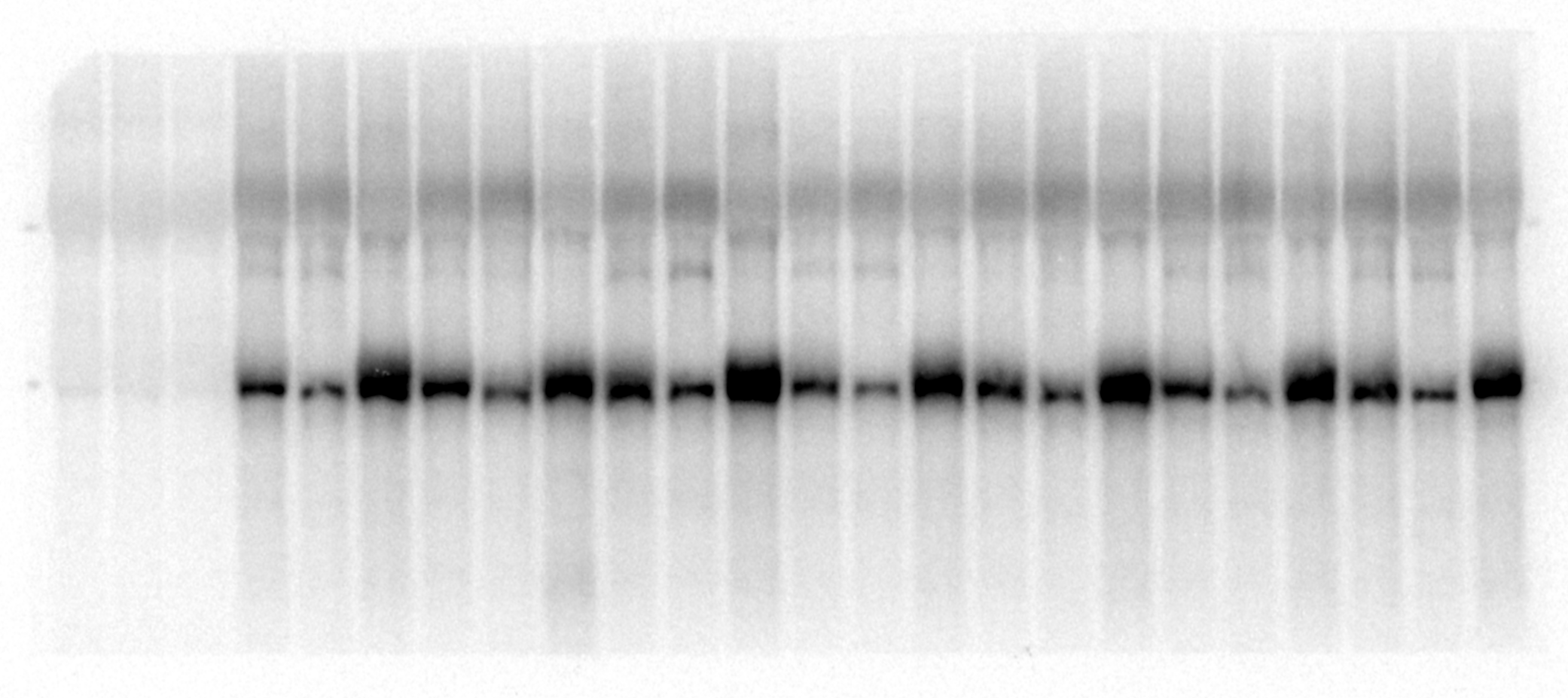

Supplement: Figure 1—source data 2. [file elife-104439-fig1-data2.zip › Figure 1-Source Data 2/Figure 1-Source Data 2.tif]

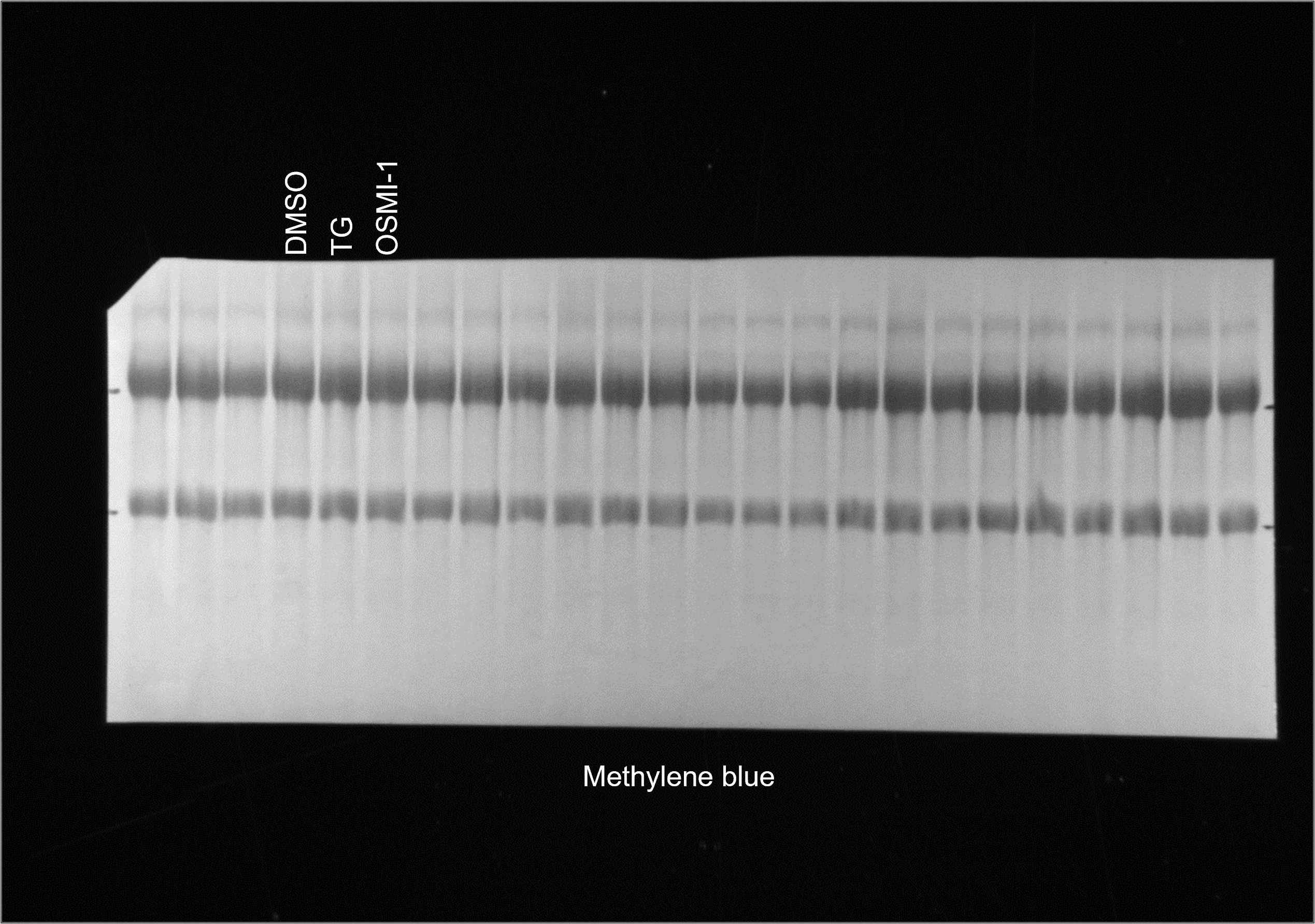

Supplement: Figure 1—source data 3. [file elife-104439-fig1-data3.zip › Figure 1-Source Data 3/Figure 1-Source Data 3-labeled.tif]

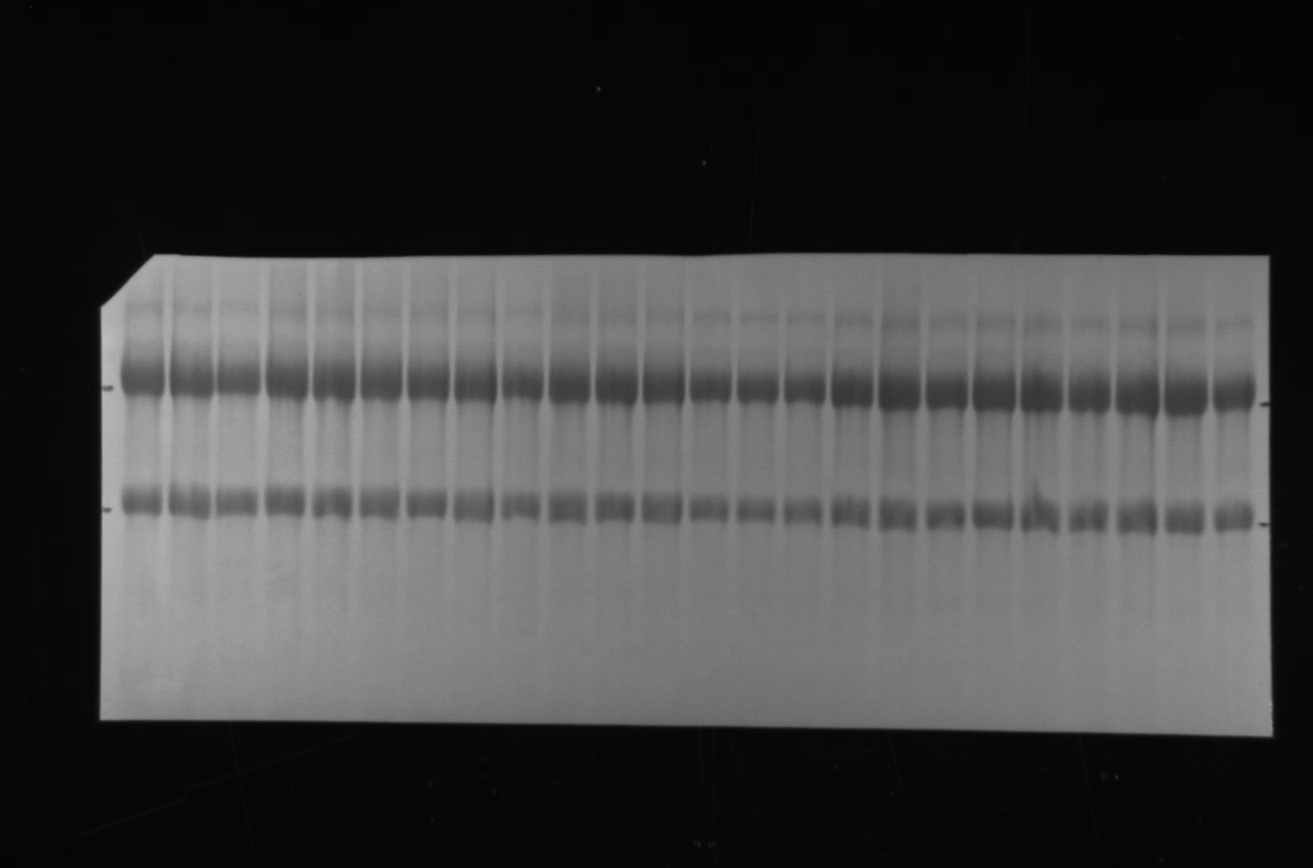

Supplement: Figure 1—source data 3. [file elife-104439-fig1-data3.zip › Figure 1-Source Data 3/Figure 1-Source Data 3.jpg]

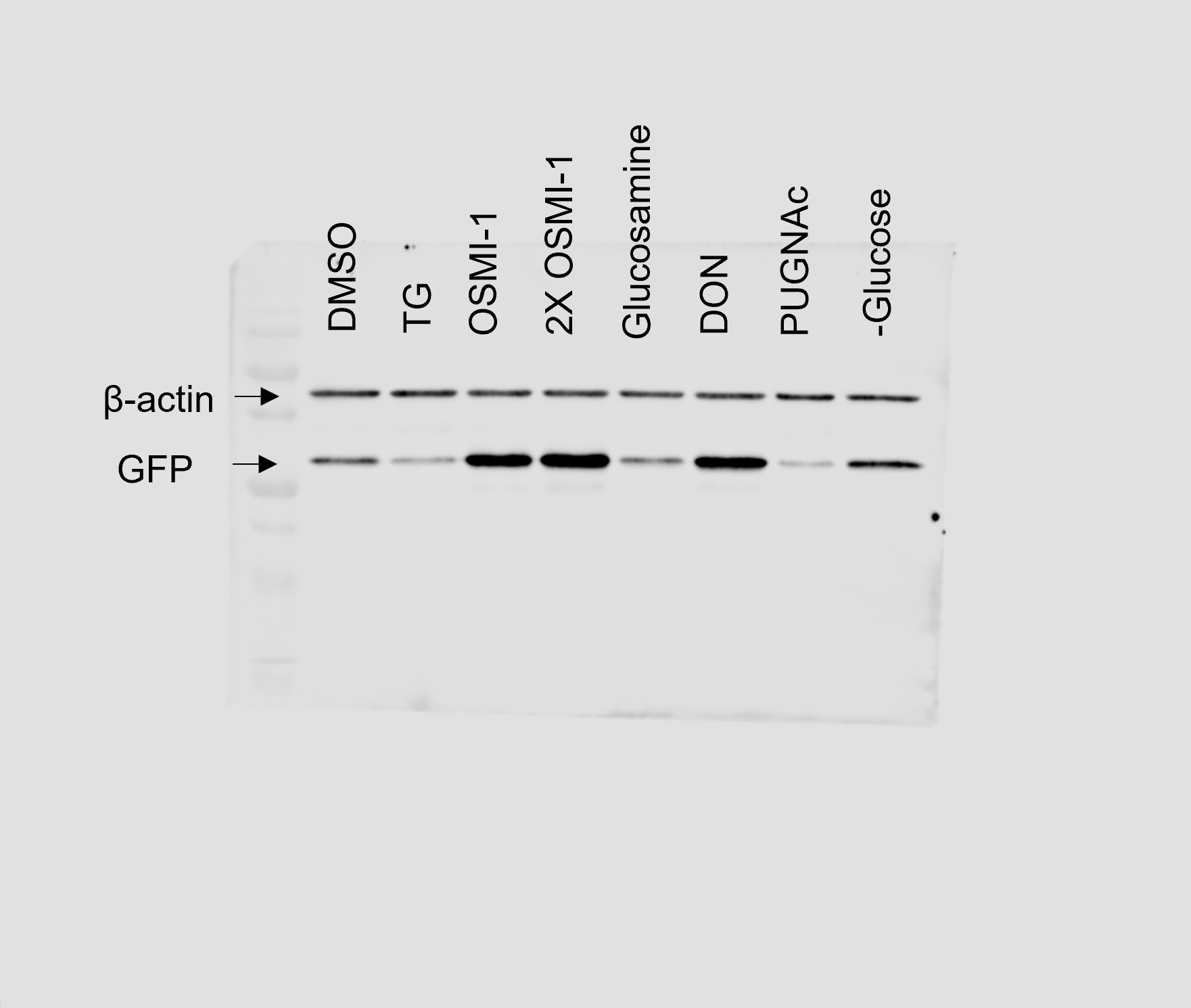

Supplement: Figure 1—source data 4. [file elife-104439-fig1-data4.zip › Figure 1-Source Data 4/Figure 1-Source Data 4-labeled.tif]

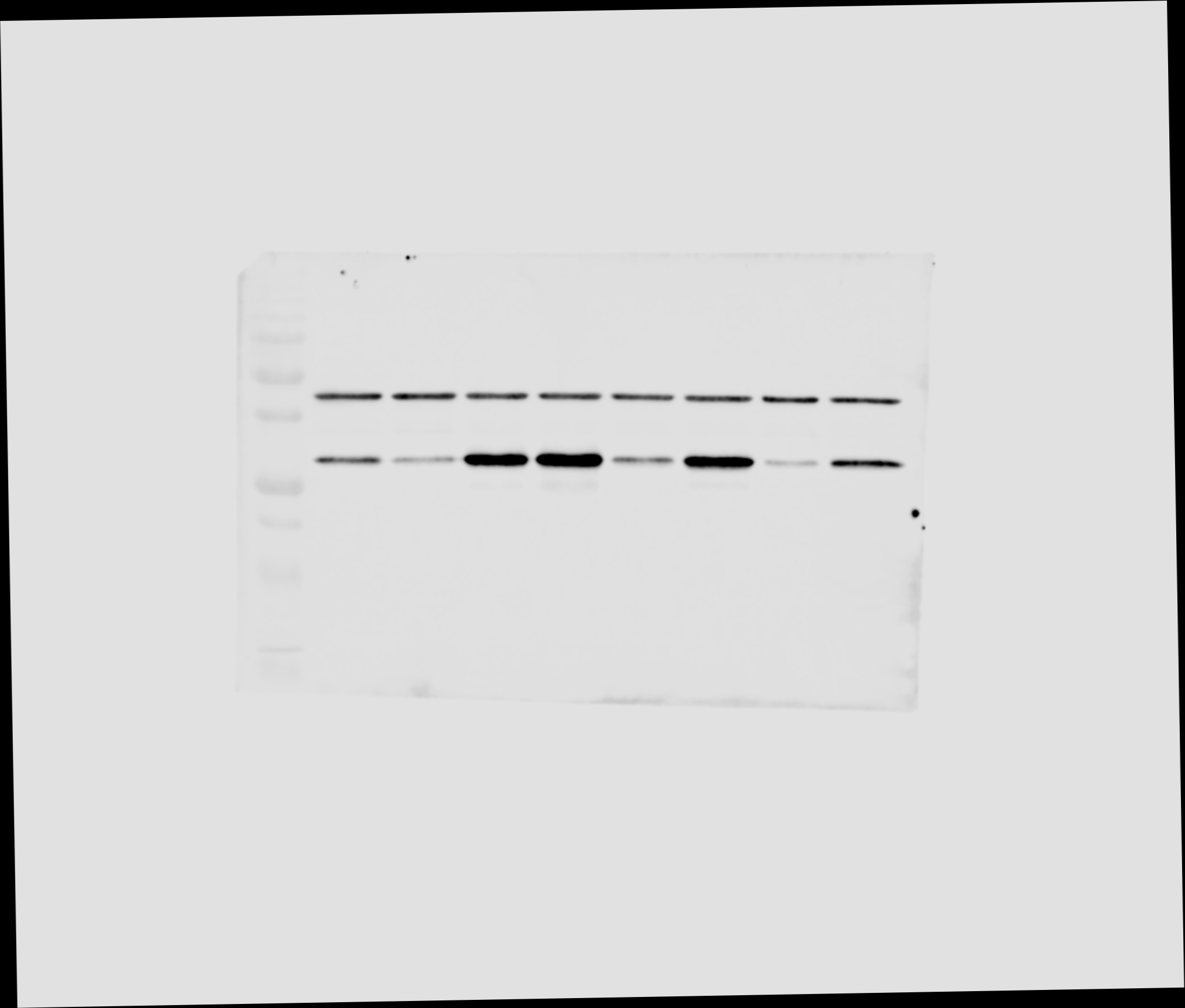

Supplement: Figure 1—source data 4. [file elife-104439-fig1-data4.zip › Figure 1-Source Data 4/Figure 1-Source Data 4.tif]

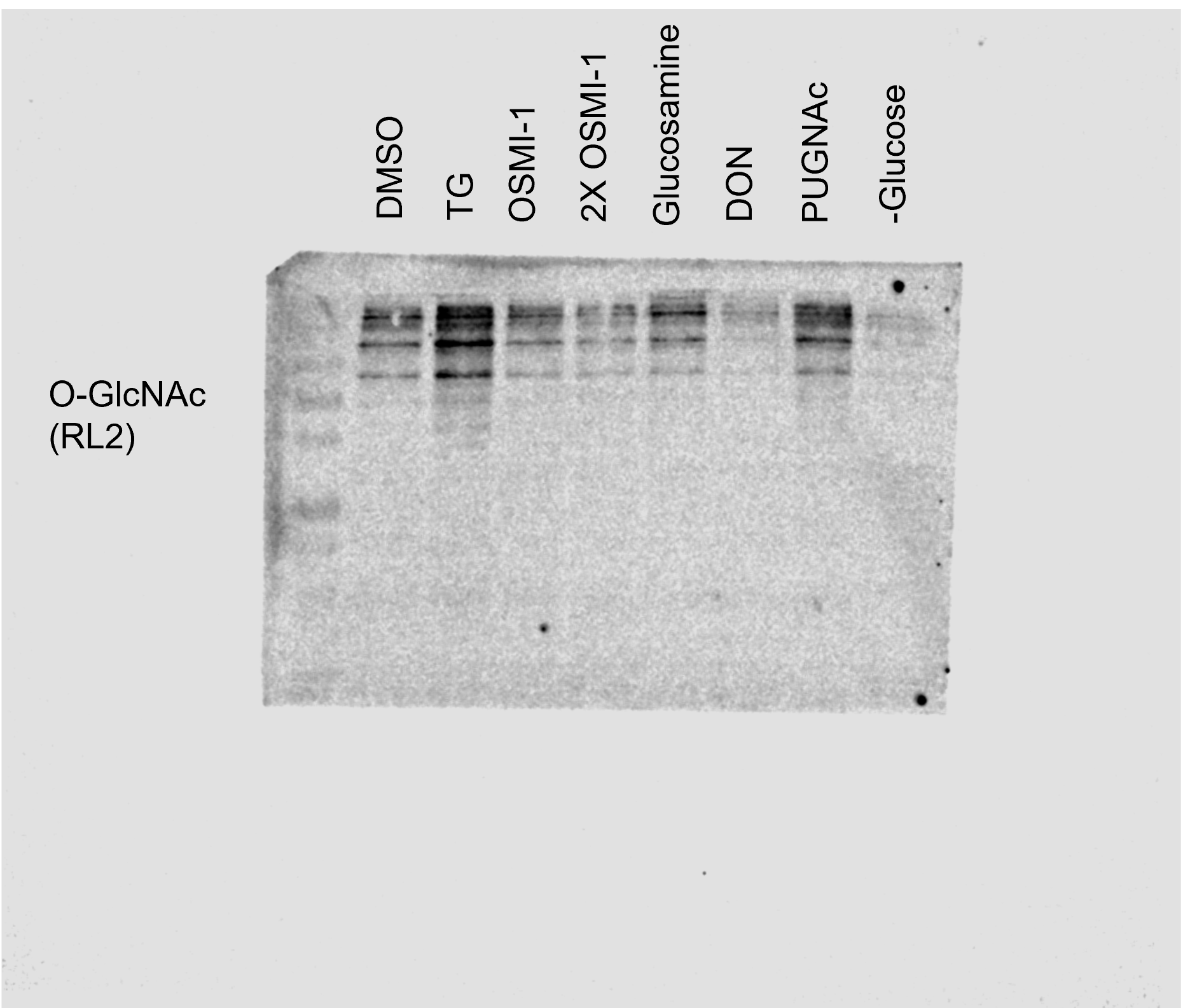

Supplement: Figure 1—source data 5. [file elife-104439-fig1-data5.zip › Figure 1-Source Data 5/Figure 1-Source Data 5-labeled.tif]

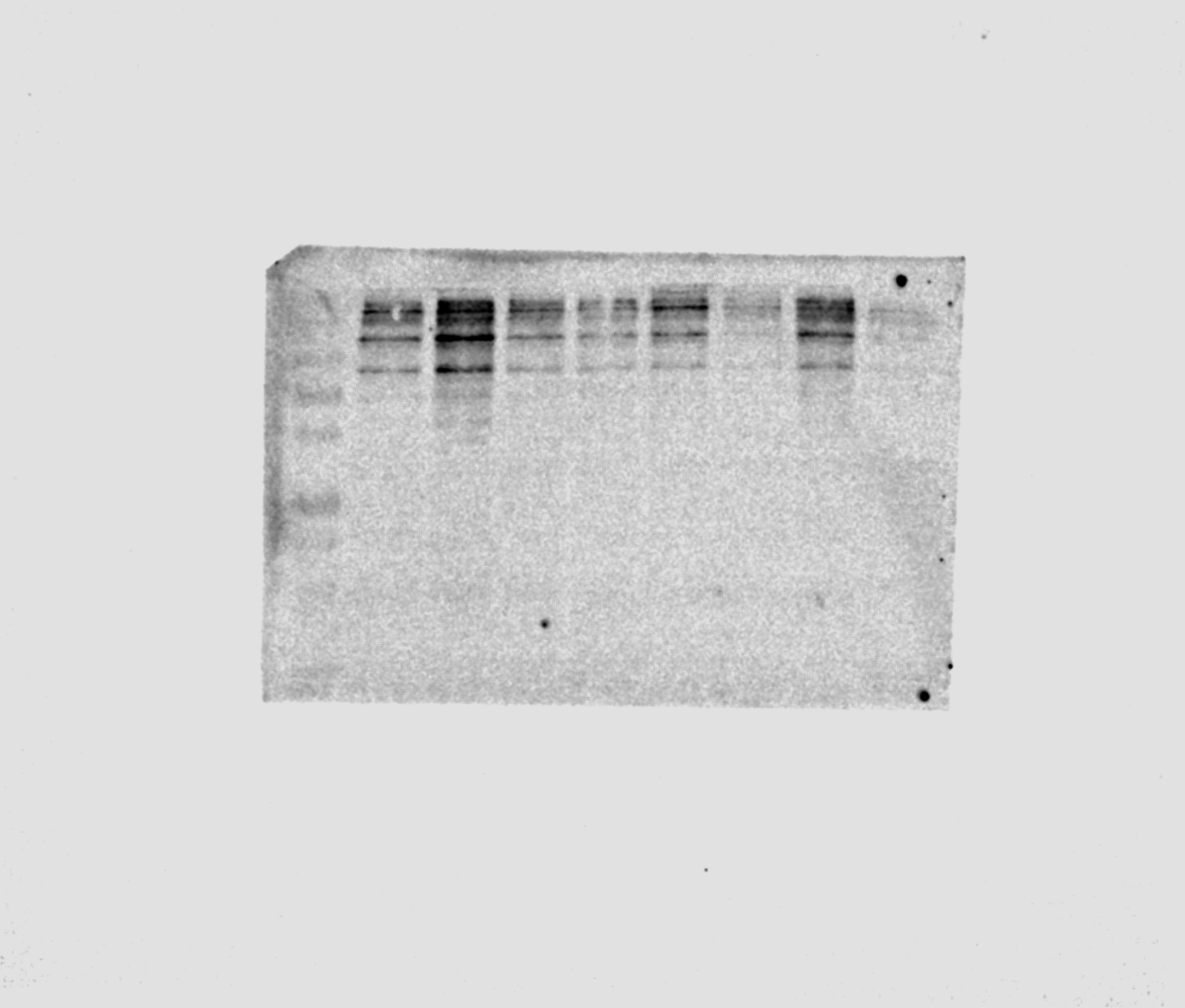

Supplement: Figure 1—source data 5. [file elife-104439-fig1-data5.zip › Figure 1-Source Data 5/Figure 1-Source Data 5.tif]

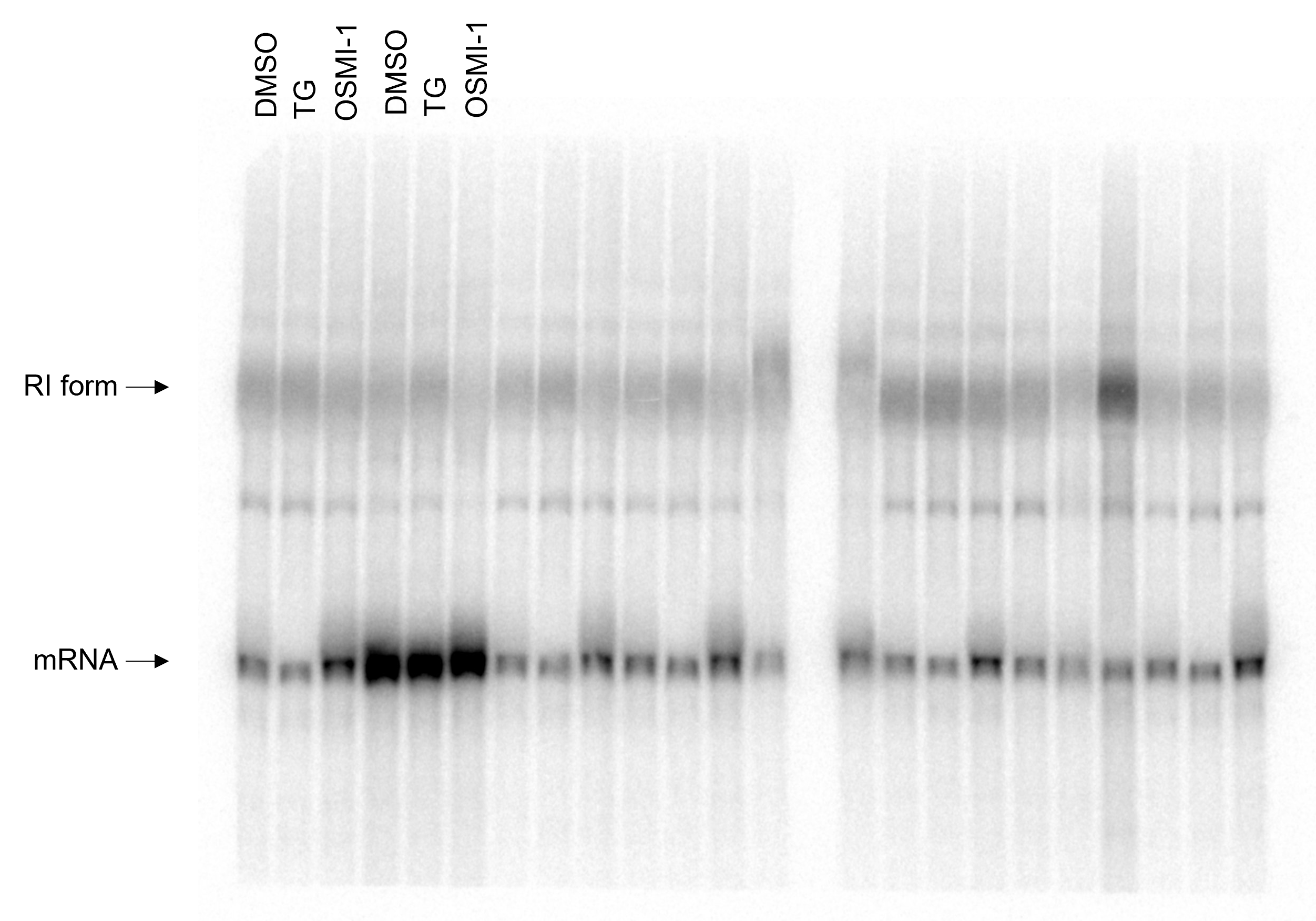

Supplement: Figure 1—source data 6. [file elife-104439-fig1-data6.zip › Figure 1-Source Data 6/Figure 1-Source Data 6-labeled.tif]

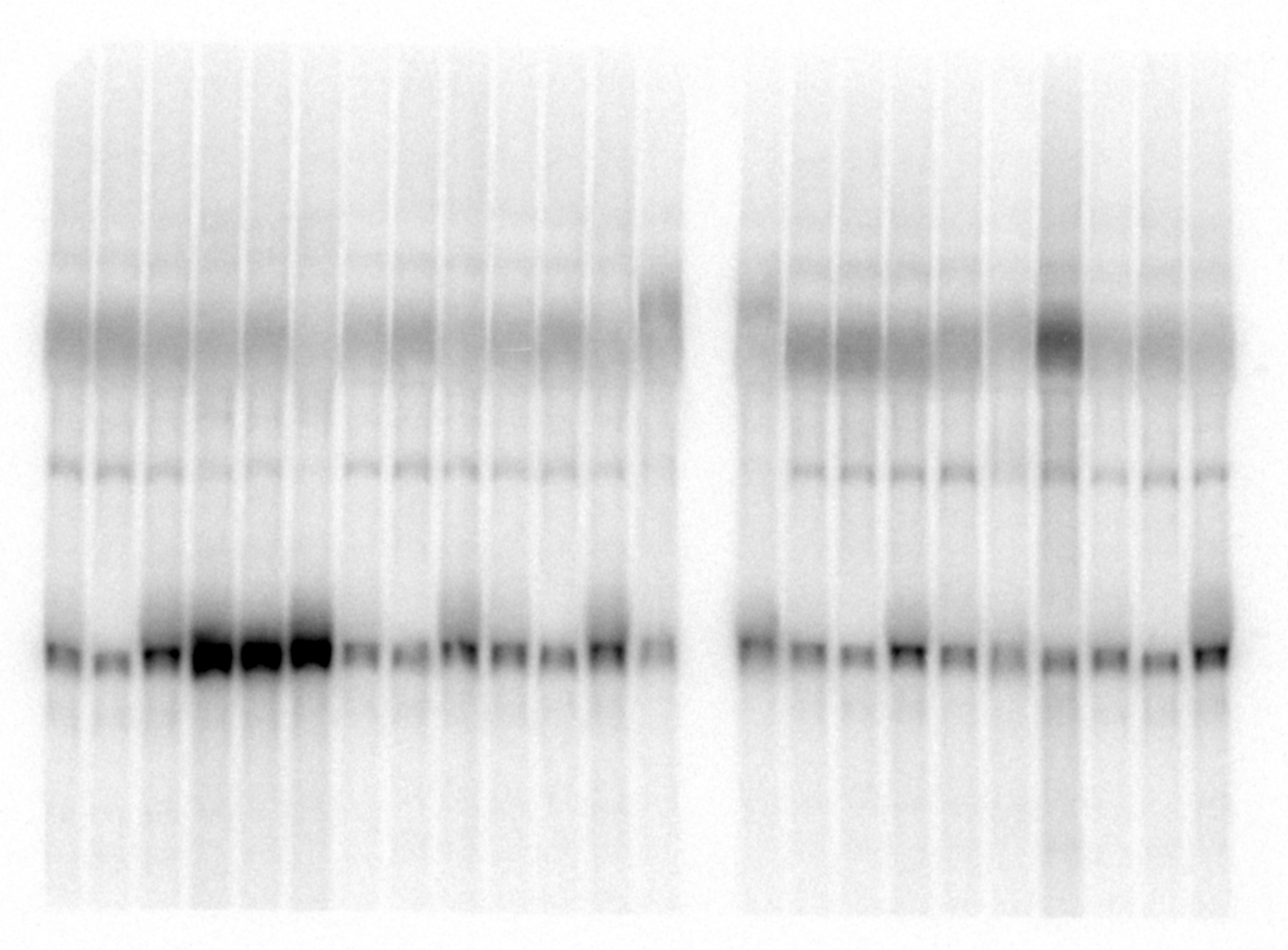

Supplement: Figure 1—source data 6. [file elife-104439-fig1-data6.zip › Figure 1-Source Data 6/Figure 1-Source Data 6.jpg]

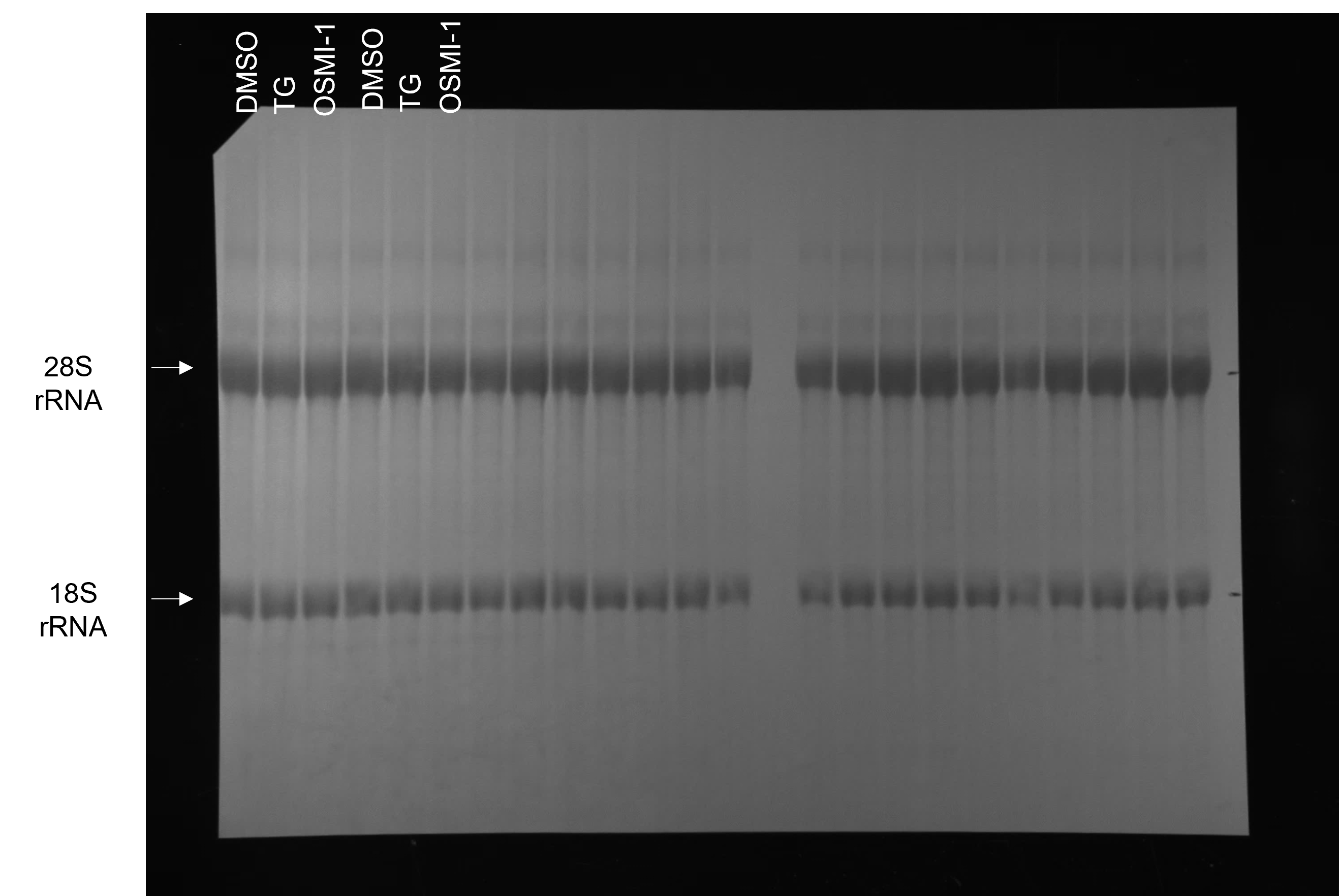

Supplement: Figure 1—source data 7. [file elife-104439-fig1-data7.zip › Figure 1-Source Data 7/Figure 1-Source Data 7-labeled.tif]

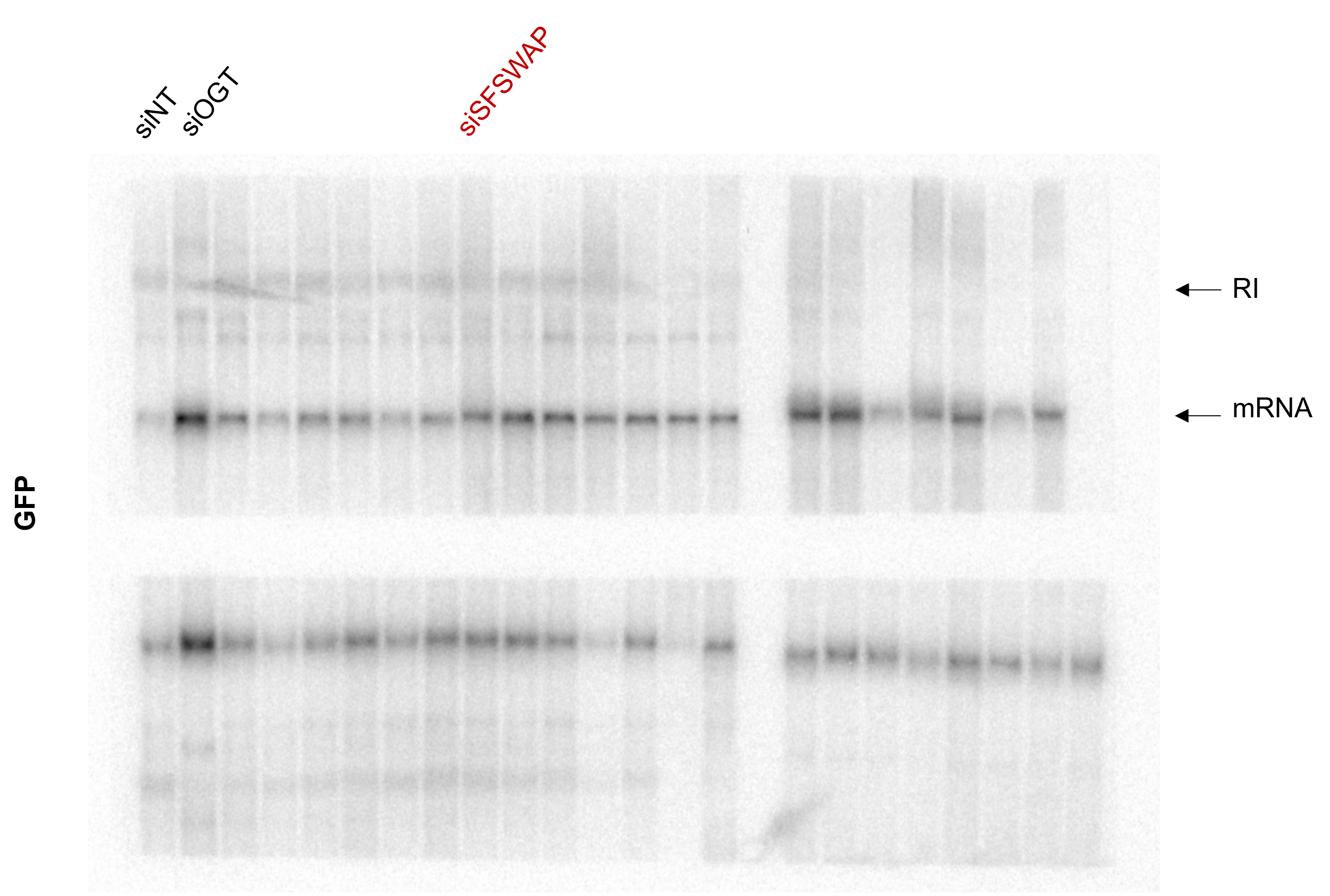

Supplement: Figure 2—source data 1. [file elife-104439-fig2-data1.zip › Figure 2-Source Data 1/Figure 2-Source Data 1-labeled.tif]

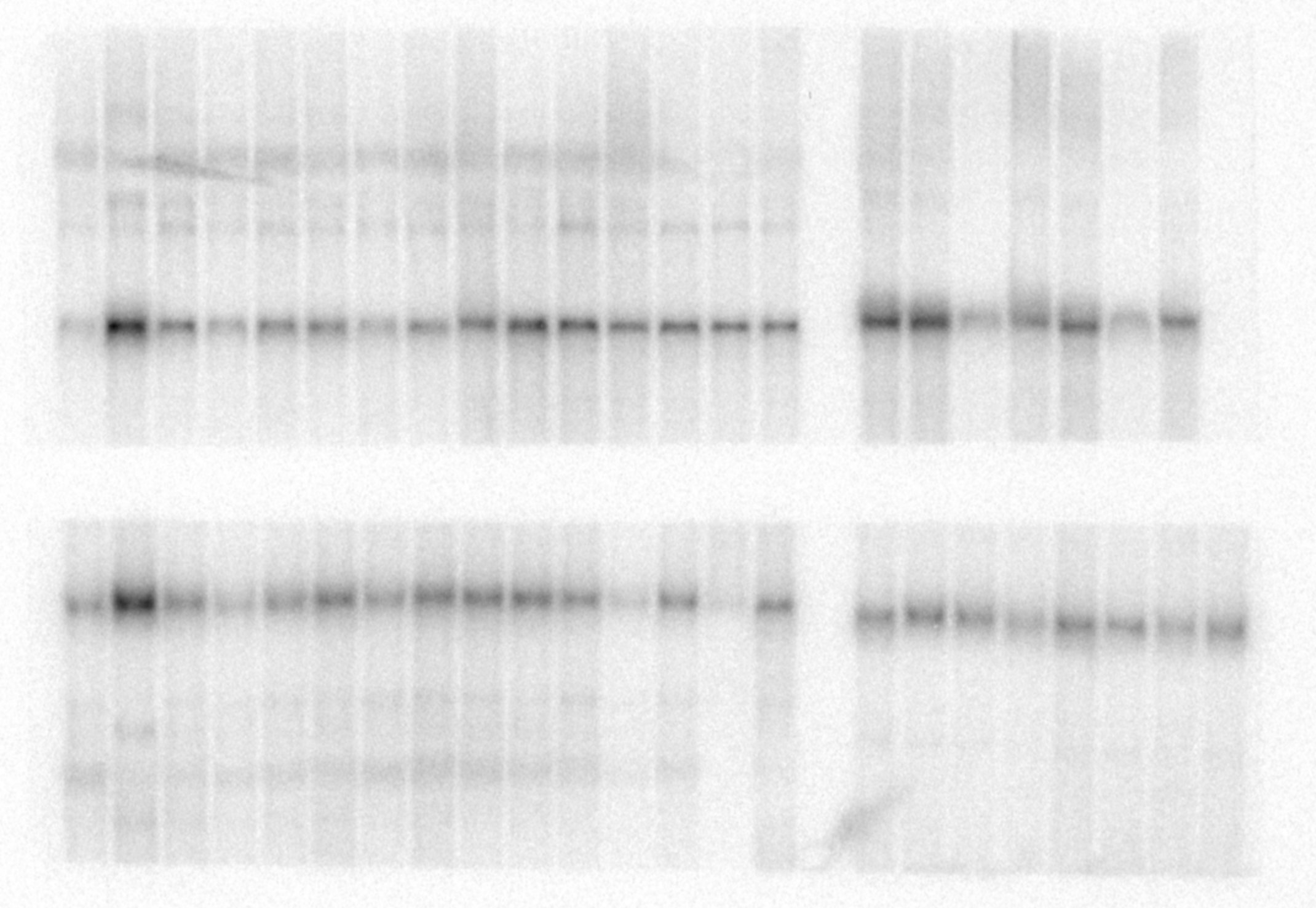

Supplement: Figure 2—source data 1. [file elife-104439-fig2-data1.zip › Figure 2-Source Data 1/Figure 2-Source Data 1.jpg]

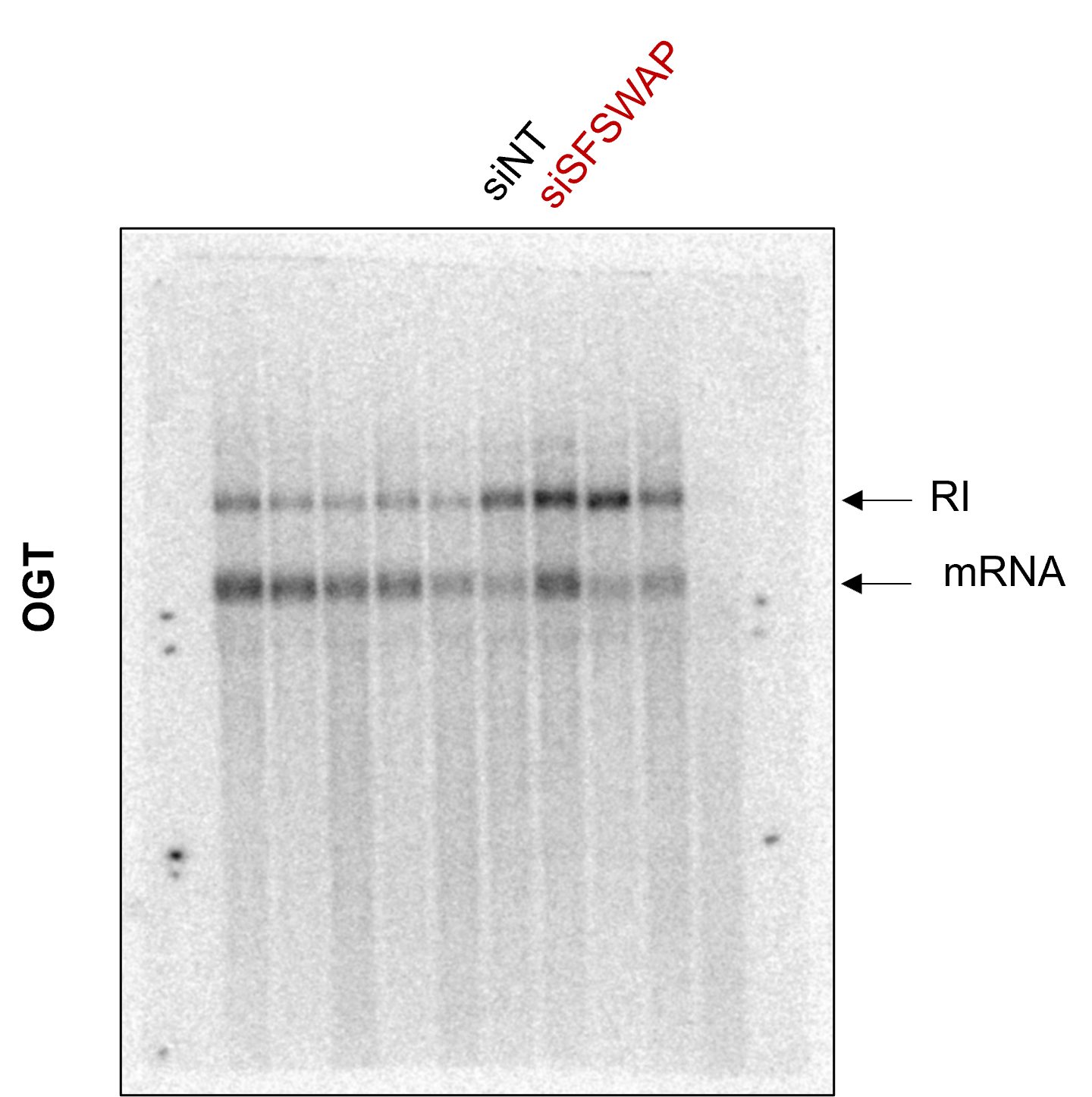

Supplement: Figure 2—source data 2. [file elife-104439-fig2-data2.zip › Figure 2-Source Data 2/Figure 2-Source Data 2-labeled.tif]

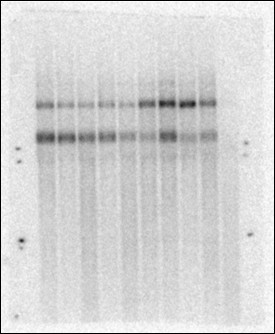

Supplement: Figure 2—source data 2. [file elife-104439-fig2-data2.zip › Figure 2-Source Data 2/Figure 2-Source Data 2.jpg]

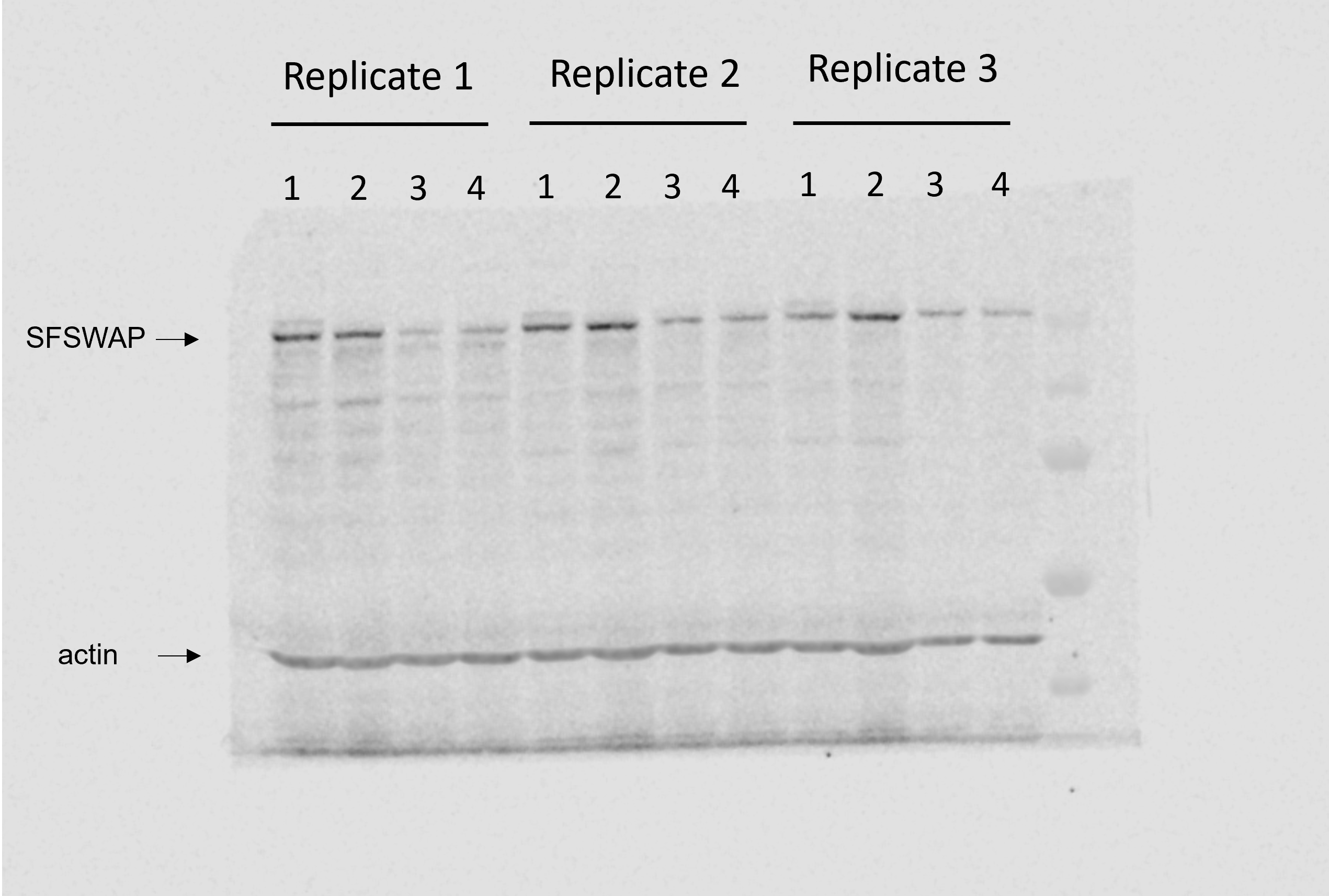

Supplement: Figure 3—figure supplement 1—source data 1. [file elife-104439-fig3-figsupp1-data1.zip › Figure 3-Figure supplement 1-Source Data 1/Figure 3-Figure supplement 1-Source Data 1-labeled.tif]

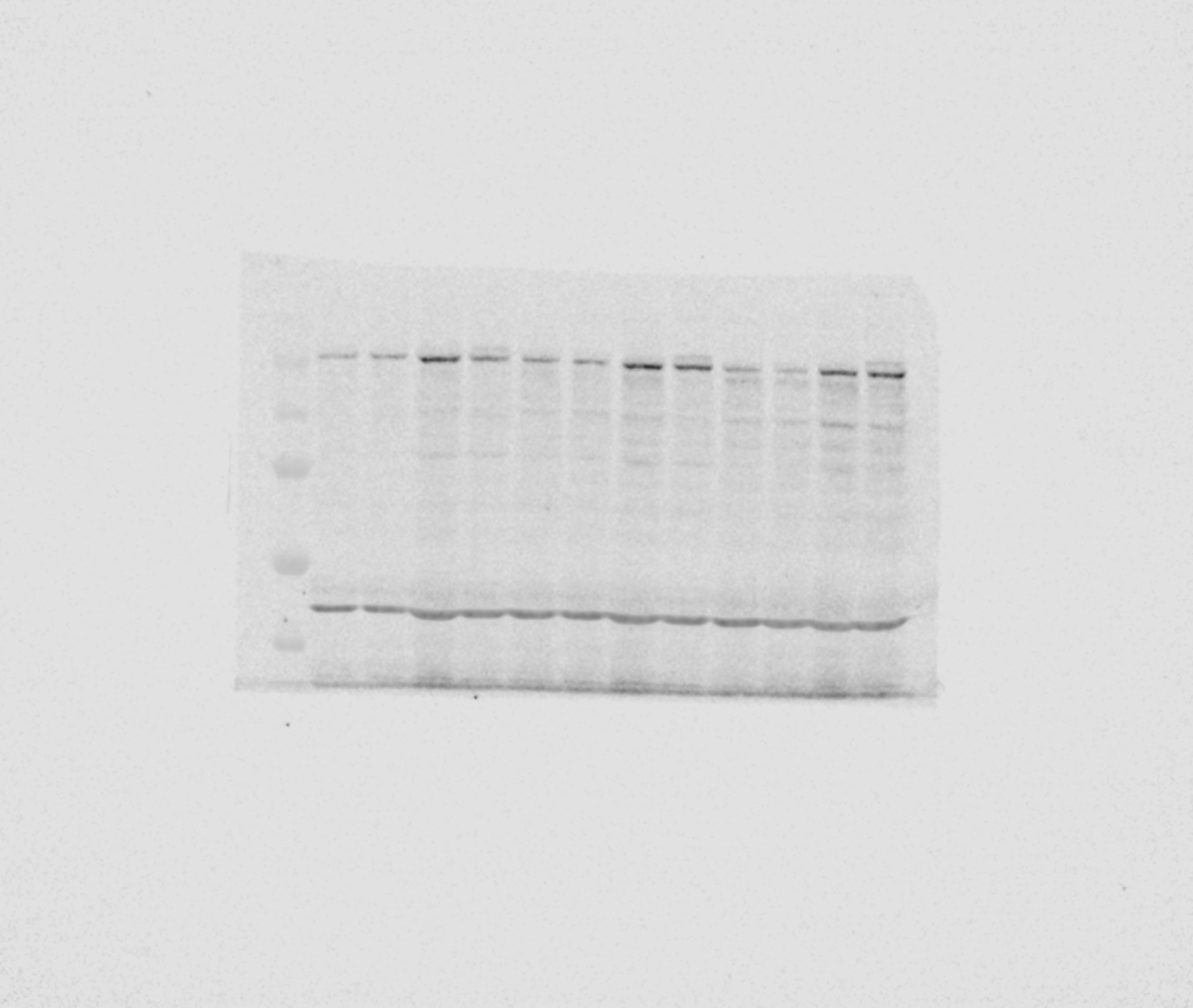

Supplement: Figure 3—figure supplement 1—source data 1. [file elife-104439-fig3-figsupp1-data1.zip › Figure 3-Figure supplement 1-Source Data 1/Figure 3-Figure supplement 1-Source Data 1.tif]

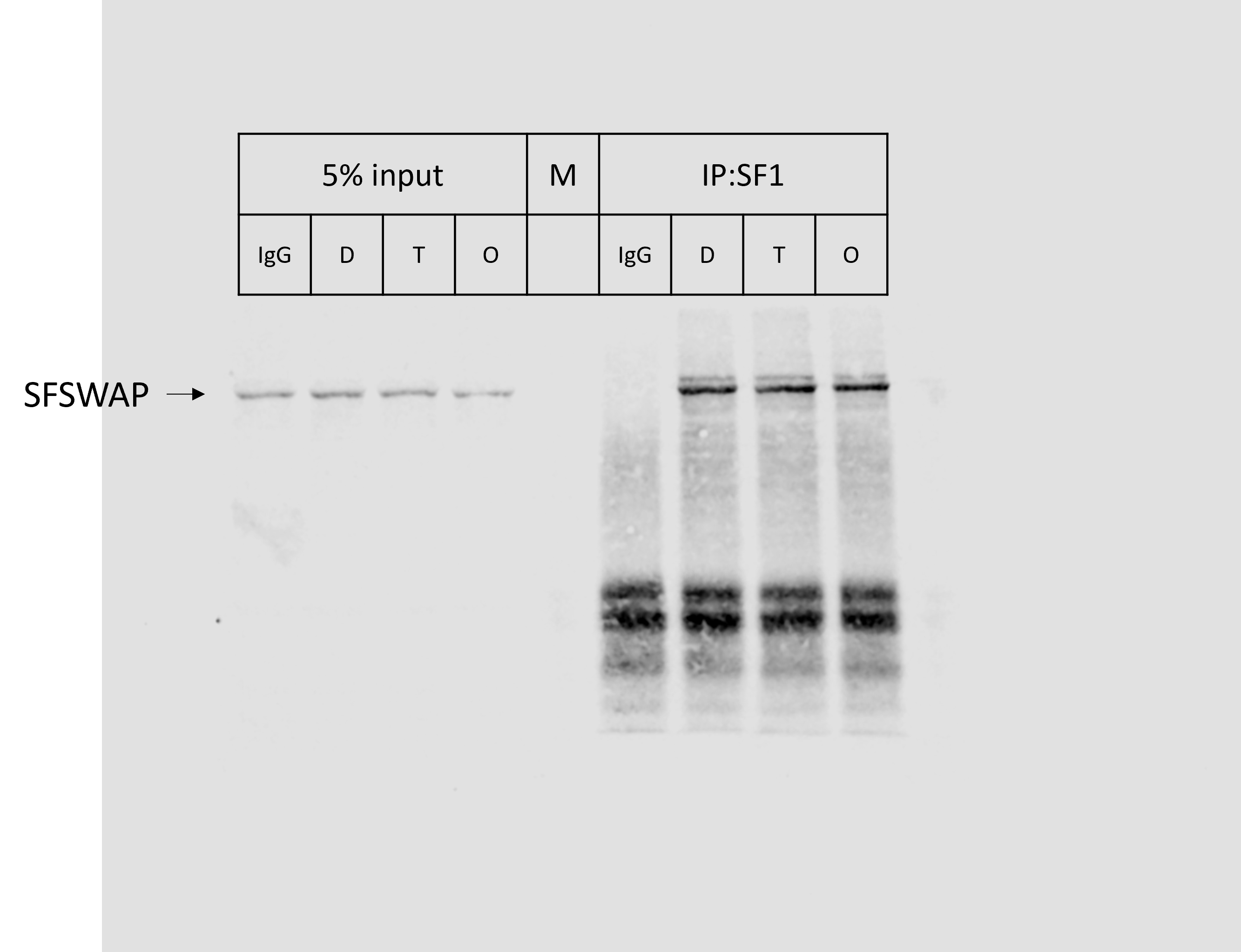

Supplement: Figure 6—figure supplement 2—source data 1. [file elife-104439-fig6-figsupp2-data1.zip › Figure 6-Figure supplement 2-Source Data 1/Figure 6-Figure supplement 2-Source Data 1-labeled.tif]

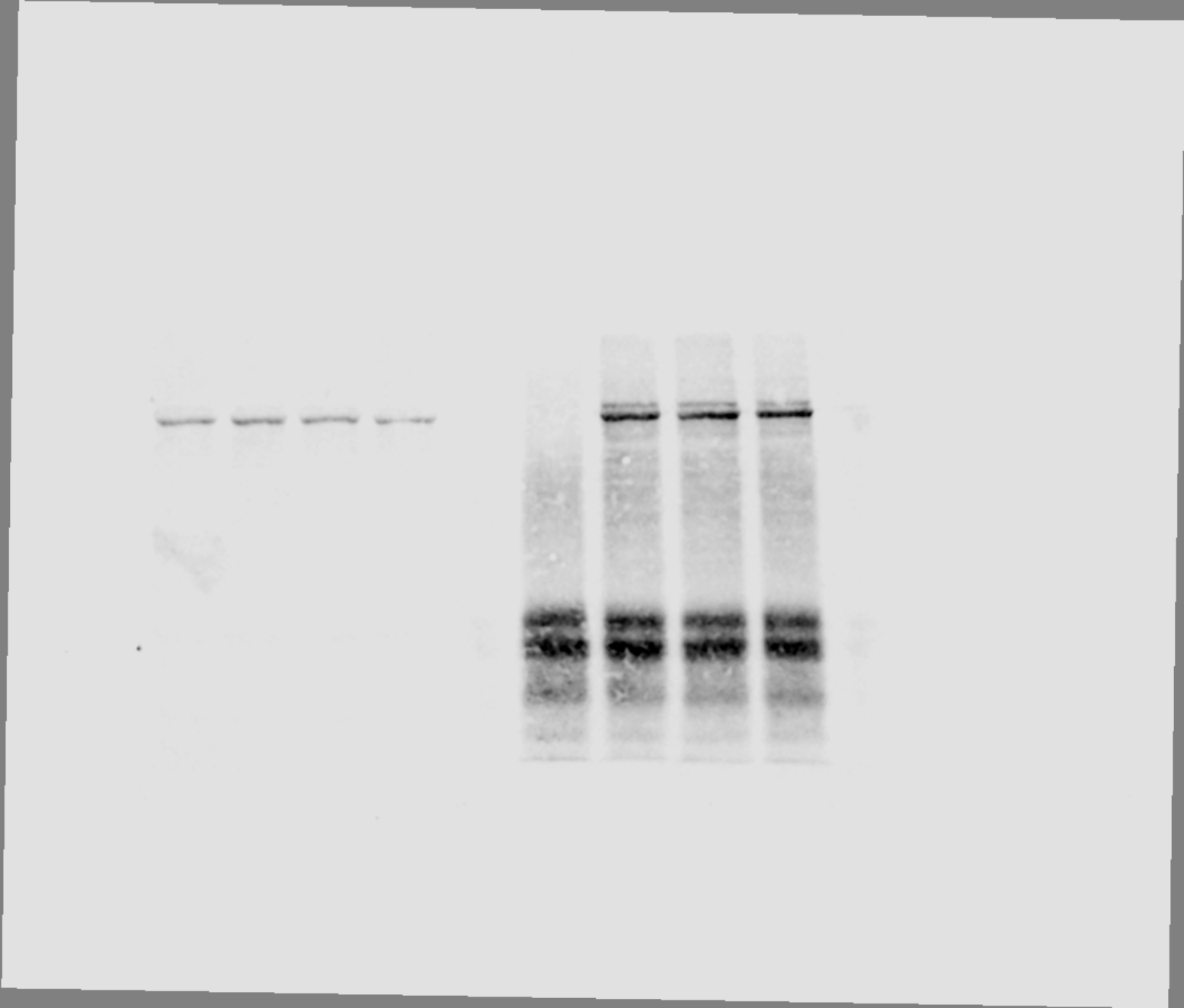

Supplement: Figure 6—figure supplement 2—source data 1. [file elife-104439-fig6-figsupp2-data1.zip › Figure 6-Figure supplement 2-Source Data 1/Figure 6-Figure supplement 2-Source Data 1.tif]
