# Supplementary material for: SFSWAP is a negative regulator of OGT intron detention and global pre-mRNA splicing
Source: eLife. 2025 Apr 23;13:RP104439. doi: 10.7554/eLife.104439 (PMC12017769; doi:10.7554/eLife.104439)
Supplement: Supplementary file 32 [file elife-104439-supp8.zip › Supplementary file 9.odt]

| Name | Sequence | Description |
| --- | --- | --- |
| NC4540 | CCAAGATTACTCCAAGCTACTG | OGT exon 5 reverse primer for RT-qPCR |
| NC4275 | CTGGGTCGCTTGGAAGAAG | OGT exon 4 forward primer for RT-qPCR |
| NC4273 | CCTTTCCCTCCCATCTTCTTTC | OGT RI forward primer for RT-qPCR |
| NC4278 | ACAACCTCCTCCTCCTCTT | OGT decoy forward primer for RT-qPCR |
| dT20 | TTTTTTTTTTTTTTTTTTTT | dT20 primer for reverse transcription |
| NC988 | CATGCATTGCGTCTCAAACCT | OGT exon 3 forward primer |
| NC1769 | TTCAGAGAGTCTGCATGGGT | OGT exon 8 reverse primer |
| NC4588 | CACAAGATCCTCATCGACAGATA | SFSWAP forward primer for RT-qPCR |
| NC4589 | CTCCTCTTCAGACAGCTGATAAT | SFSWAP reverse primer for RT-qPCR |
| NC1224 | ACCCAGCACAATGAAGATCA | β-actin forward primer for RT-qPCR |
| NC1225 | CTCGTCATACTCCTGCTTGC | β-actin reverse primer for RT-qPCR |
| NC3739 | CTTCGAATTCTGCAGTCGACGGTACCATGGTGAGCAAGGGCGAGGA | BamHI site containing reverse primer for OGT reporter cloning |
| NC3482 | GGGAAAGAAAACATCAAGGG | Acc65I site containing forward primer for OGT reporter cloning |
| NC2085 | CTGAGTGAGCTGCACTGTGA | Forward primer used in OGT reporter cloning; introduces BamHI site |
| NC3851 | GATTATGATCTAGAGTCGCGGCCGCTTTAGTGATACTTGTGGGCCA | Reverse primer used in OGT reporter cloning; introduces XbaI site |
| NC3378 | TAAACGGCCACAAGTTCAGC | Forward primer used for OGT reporter validation; binds GFP ORF |
| NC2094 | CACCAGCCACCACTTTCTGA | Reverse primer used for OGT reporter validation |
| NC985 | ACACAGCCAAGATTACTCCAAG | OGT exon 5 reverse primer used for reverse transcription |
| NC2909 | ACCACATGAAGCAGCACGAC | Forward primer used to generate GFP probe for northern blot analysis |
| NC4001 | TAATACGACTCACTATAGGGATCTTGAAGTTCACCTTGATG | Reverse primer used to generate GFP probe for northern blot analysis |
| NC2018 | CAGTGTAAATCACGGAATATC | Forward primer used to generate OGT probe for northern blot analysis |
| NC1248 | TAATACGACTCACTATAGGGAAGGATCGCAAGACAACATCT | Reverse primer used to generate OGT probe for northern blot analysis |
